# The Effect of Job Satisfaction on Psychological Well-Being for Taiwanese Home-Care Workers, Mediated by Emotional Labor

**DOI:** 10.3390/healthcare11182514

**Published:** 2023-09-11

**Authors:** Tung-Sheng Kuo, Li-Chuan Chu, Pi-Lien Kao, Chia-Lung Shih

**Affiliations:** 1 Department of Business Administration, Nanhua University, Chiayi County 622301, Taiwan; tskuo@nhu.edu.tw (T.-S.K.);; 2 General Affairs Office, Ditmanson Medical Foundation Chia-Yi Christian Hospital, Chiayi City 60056, Taiwan; 3 Clinical Research Center, Ditmanson Medical Foundation Chia-Yi Christian Hospital, Chiayi City 60056, Taiwan

**Keywords:** psychological well-being, home-care workers, job satisfaction, emotional labor

## Abstract

The objective of this study was to investigate the effect of job satisfaction on psychological well-being through emotional labor for Taiwanese home-care workers. A total of 316 home-care workers who worked in Chiayi, Taiwan, were recruited in this study. Most of the participants were Taiwanese (96.5%). The mean age of the participants was 42.05 ± 12.15 years, and the participants were predominantly female (80%). The mean work experience was 5.64 ± 5.13 years. Job satisfaction, emotional labor, and psychological well-being were measured. Partial least squares structural equation modeling was used to examine the direct and indirect effects on job satisfaction, emotional labor, and psychological well-being. The results demonstrated that the internal factors of job satisfaction had indirect effects on psychological well-being through the mediating effects of surface acting and deep acting. However, it was also observed that the external factors of job satisfaction had indirect effects on psychological well-being through the mediating effect of surface acting but not deep acting. The model explained 57.6% of the variance in psychological well-being. The internal factors of job satisfaction are more important than the external factors affecting psychological well-being through the mediating effect of deep acting. Based on our results, we recommend enhancing the deep acting of emotional labor to improve the psychological well-being of Taiwanese home-care workers.

## 1. Introduction

### 1.1. Taiwanese Home-Care Workers 

Taiwan’s aging population has grown rapidly, with the percentage of elderly people exceeding 15% in 2019 [1]. It was reported that 14.05% of elderly individuals experience difficulties with the activities of daily living [2]. Due to the increasing number of aging people and elderly people with disabilities in Taiwan, the government provided the plan (National 10-Year Long-Term Care Plan) [2,3]. In this plan, they highlighted community- and home-based services. Thus, home care workers are increasingly needed in Taiwan to provide home-based services. Home-based services include medical (such as nursing care and rehabilitation) and nonmedical services (such as toileting, showering, ambulation, cooking, and laundry). Thus, home-care workers play an important role in taking care of patients at home. The employment rate of home-care workers in Taiwan is still low (53%) [4]. To meet the growing needs of home-based services in Taiwan, we should investigate the factors that may affect the psychological well-being of home-care workers. This information could be adopted to improve their psychological well-being, and thus, provide a motivating strategy to increase the employment rate. 

### 1.2. Job Satisfaction

Job satisfaction is defined as the combination of psychological, physiological, and environmental circumstances that lead a person to honestly say, “I am satisfied with my job.” [5]. Research has reported a positive association between job satisfaction and the intention to stay among Taiwanese nurses [6]. There is a clear connection between the satisfaction that nurses feel with their jobs and the work environment in which they operate [7]. Patients’ perceptions of the overall quality of care are positively correlated with nurses’ general job satisfaction [8]. The nursing work environment has been reported to be associated with job satisfaction [9]. Job satisfaction has been reported to be related to employee withdrawal, burnout, and mental health [10,11]. It has been demonstrated that workers who are very content with their jobs are more productive, more creative, and stay with the company for which they work for a longer amount of time [12]. 

### 1.3. Psychological Well-Being

Psychological well-being is manifested through a sense of wellness [13]. Older adults who have higher levels of psychological well-being tend to have a lower risk of mortality [13]. The effectiveness of a person’s total psychological functioning is typically used to define psychological well-being [14]. Clinical psychologists demonstrated that the pleasantness dimension of well-being could affect various individual outcomes. For example, people who are depressed have very low self-esteem, have a pessimistic outlook, are less motivated, and think more slowly [15,16]. Previous studies have demonstrated that psychological well-being is associated with worker performance [17]. Research shows that teachers who are satisfied with their jobs tend to have higher levels of well-being [18]. To the best of our knowledge, no study has demonstrated this association among Taiwanese home-care workers.

### 1.4. Emotional Labor

Emotional labor is considered a key factor in nursing and may have a high influence on workers and their organization. Emotional labor is regarded as a crucial aspect of the healthcare professional’s role [19]. Emotional labor is the process of dealing with expressions and feelings to meet a job’s emotional requirements. Thus, emotional labor plays a significant role in the job of home-care workers when they interact with their patients. Two primary regulation strategies comprise emotional labor, including surface acting and deep acting. Surface acting indicates that the employees attempt to mask their actual emotions when dealing with their patients. In contrast, deep acting is the process in which employees adjust their emotions to fit their expected role. Emotional labor among workers in care work and other service jobs was reported to cause adverse work outcomes, such as burnout or low job satisfaction [20,21]. Emotional labor has been reported to be associated with burnout for teachers [22]. Based on the above discussion, we assume that job satisfaction impacts psychological well-being through the mediating effect of emotional labor.

### 1.5. Objective and Hypotheses

Due to the growing need for home-based services in Taiwan, it is critical to investigate the factors that may affect home-care worker’s psychological well-being. Based on a review of previous studies, the following hypotheses were generated:

**H1.**  *External factors of job satisfaction are positively related to surface acting.*

**H2.**  *External factors of job satisfaction* *are positively related to deep acting.*

**H3.**  *Internal factors of job satisfaction are positively related to surface acting.*

**H4.**  *Internal factors of job satisfaction are positively related to deep acting.*

**H5.**  *Surface acting is positively related to psychological well-being.*

**H6.**  *Deep acting is positively related to psychological well-being.*

**H7.**  *External factors of job satisfaction are positively related to psychological well-being.*

**H8.**  *Internal factors of job satisfaction are positively related to psychological well-being.*

**H9.**  *Internal factors of job satisfaction are positively and indirectly related to psychological well-being through surface acting.*

**H10.**  *Internal factors of job satisfaction are positively and indirectly related to psychological well-being through deep acting.*

**H11.**  *External factors of job satisfaction are positively and indirectly related to psychological well-being through surface acting.*

**H12.**  *External factors of job satisfaction are positively and indirectly related to psychological well-being through deep acting.*

Based on the above hypotheses, the objectives of this study are as follows:

(1) To investigate the association between job satisfaction and emotional labor and to compare whether the internal factors of job satisfaction associated with emotional labor have a greater effect than the external factors of job satisfaction.
(2) To investigate the association between emotional labor and psychological well-being.
(3) To investigate whether the effect of job satisfaction on psychological well-being is mediated by emotional labor.

We assumed that the effect of internal factors of job satisfaction was a more important variable than the effect of external factors on psychological well-being through the mediating effect of emotional labor. We expected that our results could provide valuable information to improve the low employment rate of home-care workers and increase the quality of home services.

## 2. Materials and Methods

### 2.1. Study Design and Participants

This was a descriptive cross-sectional study. This study has been approved by the Institutional Review Board (IRB) of Ditmanson Medical Foundation Chia-Yi Christian Hospital (IRB No.: IRB2022050). The participants were the home-care workers who work in Chiayi, Taiwan. The inclusion criteria are as follows: (1) home-care workers with work experience for 6 months or more; (2) home-care workers who were not directly involved in patient care (e.g., managers); and (3) home-care workers aged from 20 to 65 years old. Part-time home-care workers (weekly working hours ≤ 2.5 h) were excluded. The questionnaire included participants’ characteristics, emotional labor, job stratification, and psychological well-being. These measures are described in detail in the following sections.

### 2.2. Measures

Participants’ characteristics were collected, including nationality, age, sex, marital status, education level, work experience, and monthly salary. The English questionnaires were translated into Chinese because the measures we chose were in English. A 5-point Likert scale (1 = strongly disagree to 5 = strongly agree) was used to measure each item. Psychological well-being was assessed based on the method by Hills et al. [23] and a total of 14 items were adopted for assessment. Job satisfaction was assessed based on a previous study [24]. Job satisfaction included 16 items and these could be classed as internal (8 items) and external (8 items) factors (Appendix A). Emotional labor was assessed by using those items developed by Diefendroff et al. [25]. Emotional labor was divided into surface acting and deep acting. The surface acting questionnaire included four items which indicate the regularity with which they pretended to feel things when providing service delivery. The deep acting questionnaire also included four items which indicated that workers altered their emotions to show sincere expressions. To determine the score for each domain, we added up the scores of the related items.

### 2.3. Statistical Analysis

All statistical analyses were performed using SPSS version 23.0. Differences in continuous or categorical variables between two groups were estimated by using a two-sample t-test. Differences in continuous variables among more than two groups were estimated using one-way ANOVA (analysis of variance), followed by the Tukey test. Statistical analysis was performed with a confidence level of 95%. We used the equation mentioned by Hajji et al. (2023) to calculate the effect sizes [26]. A value of 0.01 indicates a small effect, 0.06 indicates a moderate effect, and 0.14 indicates a large effect [27]. A *p*-value less than 0.05 was considered statistically significant for all the analyses. The reliability of the questionnaire was assessed by using Cronbach’s α. The correlation between two variables was estimated by using the Pearson’s correlation analysis. Partial least squares structural equation modeling (PLS-SEM) was used to estimate the direct and indirect effects among job satisfaction, emotional labor, and psychological well-being, and the analyses were performed using SmartPLS 4 software. The model was performed in two stages. In the first stage, the reliability and validity of the model’s constructs were evaluated. Three parameters were used to assess the reliability, convergent validity, and composite reliability of the reflective measurement model, including Cronbach’s α, the average variance extracted (AVE), and the composite reliability (CR). In the second stage, the path coefficient was calculated, and its statistical significance was tested using the Monte Carlo method in which 5000 bootstrap samples were used for this purpose. There are several reasons why we chose to use PLS-SEM for analysis. For one, it is able to assume that there are chain relationships between the items in question, which allows us to build a conceptual relationship model [28]. Additionally, we can test the model statistically to ensure its robustness. Moreover, the PLS-SEM technique is particularly flexible when it comes to data allocation and is well-suited for small sample sizes [29]. Finally, the data does not follow a normal distribution when using PLS-SEM.

## 3. Results

### 3.1. Major Characteristics

A total of 350 questionnaires were distributed to home-care workers, of which 316 met the criteria. The major characteristics of the participants are shown in Table 1. Most of the participants were Taiwanese (96.5%). The mean age of the participants was 42.05 ± 12.15 years, and the participants were predominantly female (80%). Most of the participants were married (64%). The education levels of the participants ranged from a high school to university (92%) education. Most of the participants had a monthly salary in the range of TWD 25,000–45,000 (87%). The mean work experience was 5.64 ± 5.13 years. 

### 3.2. Major Characteristics Affecting Psychological Well-Being

We investigated whether the major characteristics of the participants would affect their psychological well-being. The participants’ nation did not affect their total score for psychological well-being (*p* = 0.423). No significant difference was observed between sexes (*p* = 0.14). Age was positively correlated with psychological well-being (*r* = 0.152; *p* = 0.007). It was found that marital status had a significant effect on psychological well-being (*p* < 0.001). Married and widowed participants had significantly higher scores for psychological well-being than unmarried participants. The education levels did not affect psychological well-being. The work experience was positively correlated with the score of psychological well-being (*r* = 0.114, *p* = 0.042). The monthly working hours significantly affected psychological well-being (*p* = 0.015; effect size = 0.0006). However, no significant difference in monthly working hours was observed when using the Turkey test. The monthly salary significantly affected the psychological well-being (*p* = 0.002). The monthly salary between 35,000–45,000 TWD had a higher score than that between TWD 25,000–35,000 and less than 25,000 TWD. These findings indicate that marital status, work experience, monthly working hours, and monthly salary could be the confounding factors for psychological well-being. 

### 3.3. Job Satisfaction Associated with Psychological Well-Being 

We investigated the association between job satisfaction and psychological well-being using the Pearson correlation analysis. The internal factors of job stratification were positively correlated with psychological well-being (*r* = 0.631, *p* < 0.001) and its subscales (*r* = 0.483, *p* < 0.001 for self-assurance; *r* = 0.385, *p* < 0.001 for life satisfaction; and *r* = 0.634, *p* < 0.001 for job achievement) (Table 2). The external factors of job satisfaction were also positively correlated with psychological well-being (*r* = 0.603, *p* < 0.001).

### 3.4. Emotional Labor Associated with Psychological Well-Being

We investigated the association between emotional labor and psychological well-being using the Pearson correlation analysis. Surface acting was positively correlated with psychological well-being (*r* = 0.507, *p* < 0.001) and its subscales (*r* = 0.466, *p* < 0.001 for self-assurance; *r* = 0.326, *p* < 0.001 for life satisfaction; and *r* = 0.455, *p* < 0.001 for job achievement) (Table 2). Deep acting was also positively correlated with psychological well-being (*r* = 0.491, *p* < 0.001) (Table 2).

### 3.5. Assessment of Structural Equation Model 

**Figure 1:**
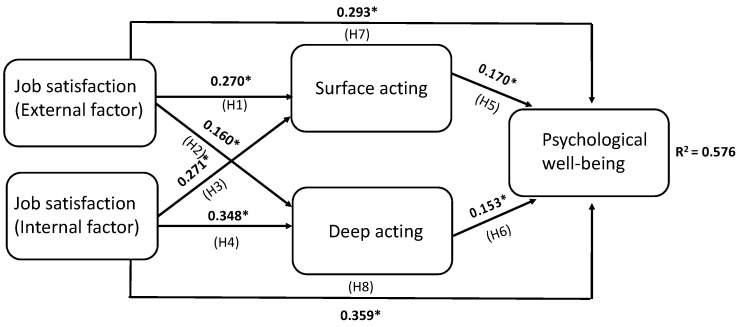
Standardized coefficients of direct effects among job satisfaction, emotional labor, and psychological well-being. *: statistical significance.

The conceptual model is presented in Figure 1, and the use of PLS-SEM to test our hypotheses is shown in Figure 1 and Table 3 and Table 4. To verify the directional relationship chain, the model’s reliability and validity are assessed using Cronbach’s alpha (≥0.70) and the loadings (>0.20) (Table 3). Good reliability and validity are indicated by these values. The reflective measurement model’s convergent validity and combined reliability are assessed using AVE (≥0.50) and CR (≥0.70), respectively (Table 3). Good convergent validity and combined reliability are indicated by these values. Our study found that the external factors of job satisfaction have direct positive effects on both surface acting (H1) and deep acting (H2) (Figure 1). Additionally, the internal factors of job satisfaction have direct positive effects on both surface acting (H3) and deep acting (H4) (Figure 1). We also found that both types of acting, surface (H5, H7) and deep (H6, H8), have direct positive effects on psychological well-being (Figure 1).

Regarding indirect effects, our results support H9, which suggests that the internal factors of job satisfaction have indirect positive effects on psychological well-being through the mediating effects of surface acting. Similarly, H10 is supported, indicating that the internal factors of job satisfaction have indirect positive effects on psychological well-being through the mediating effects of deep acting.

However, H11, which suggests that the external factors of job satisfaction have indirect positive effects on psychological well-being through the mediating effects of deep acting, is not supported by our results (Table 4). Instead, we found support for H12, which suggests that the external factors of job satisfaction have indirect positive effects on psychological well-being through the mediating effects of surface acting (Table 4). Overall, our model explains 57.6% of the variance in psychological well-being (Figure 1).

## 4. Discussion 

Due to the growing need for home-based services in Taiwan, it is critical to improve the low employment rate of home-care workers and increase the quality of home services. This study investigated the associations among job satisfaction, emotional labor, and psychological well-being. We proposed 12 hypotheses, and 11 of them were supported by our results. These hypotheses are discussed in the following sections.

### 4.1. Sample Size and Reliability

We did not calculate the required sample size for performing PLS-SEM before conducting this study. PLS-SEM was used to examine the effect of job satisfaction on psychological well-being through the mediating effect of emotional labor. In our model, a total of 8 arrows were considered. The required minimum sample size for eight arrows in the model has been suggested to be 84 [30]. A total of 316 participants were included in our model, and the sample size was large enough for performing PLS-SEM. Therefore, the results obtained from PLS-SEM should be considered reliable. Otherwise, the estimated composite reliability was larger than the generally accepted score of 0.70 for all latent variables [28]. The average variance extracted was larger than 0.5 for all latent variables. The Cronbach’s α was larger than 0.70 for all dimensions, indicating that the scale and each dimension exhibit strong reliability [31].

### 4.2. Job Satisfaction Associated with Emotional Labor

Emotional labor is considered a key factor in the workplace and can lead to high levels of effectiveness for both workers and their organizations. Emotional labor should play a significant role in the job of home-care workers when they interact with their patients. Two primary regulation strategies comprise emotional labor, including surface acting and deep acting. Job satisfaction can be classified into external and internal factors. The association between job satisfaction and emotional labor should be further investigated. In this study, we investigated this direct association among Taiwanese home-care workers. Our results demonstrated that the external factors of job satisfaction were associated with both surface acting and deep acting. These associations were also found in the internal factor of job satisfaction. A previous report indicated that wearing a false face significantly reduces job satisfaction for social workers [21]. Our results show that deep acting was more strongly related to the internal factors of job satisfaction than to the external factors. However, surface acting demonstrated a similar correlation between the internal or external factors of job satisfaction. These results show that deep acting plays a more important role in emotional labor than surface acting for Taiwanese home-care workers. 

### 4.3. Emotional Labor Associated with Psychological Well-Being

Psychological well-being is demonstrated via the existence of wellness. Previous studies have shown that psychological well-being is linked to the worker’s job performance [32]. Thus, psychological well-being should play an important role in the performance of Taiwanese home-care workers. However, no study has investigated the relationship between psychological well-being and emotional labor. Based on the Pearson correlation analysis, both surface acting and deep acting were found to be associated with psychological well-being, with a moderate strength of association (*r* = 0.491~0.507). Our results obtained using PLS-SEM demonstrated that both surface acting and deep acting were directly associated with psychological well-being, with a small strength of association (*r* = 0.153~0.170). We anticipated that deep acting would have a greater impact on psychological well-being than surface acting. However, our findings did not confirm this hypothesis, despite the fact that the correlation for surface acting was slightly stronger than that for deep acting. Emotional labor has been reported to be associated with mental health in which surface acting was negatively associated with mental health and deep acting was positively associated with mental health [33]. This result could support our findings among Taiwanese home-care workers. 

### 4.4. Job Satisfaction Associated with Psychological Well-Being

Job satisfaction has been demonstrated to have a positive relationship with psychological well-being [18]. This study provides further evidence of this association among Taiwan home-care workers. Our results, obtained from PLS-SEM, demonstrate that both the internal and external factors of job satisfaction were positively associated with psychological well-being (Figure 1). Based on the correlation coefficients, the internal factors of job satisfaction (*r* = 0.359) appear to have a more significant impact on psychological well-being than the external factors of job satisfaction (*r* = 0.293). As expected, job satisfaction is positively correlated with psychological well-being. Furthermore, both the internal and external factors of job satisfaction were positively correlated with psychological well-being. The internal factors affecting job satisfaction are related to psychological satisfaction, indicating that the internal factor of job satisfaction may play a more important role in promoting psychological well-being than the external factors. A previous report also demonstrated a strong correlation between job satisfaction and psychological well-being for preventive medicine workers [34].

### 4.5. Effect of Job Satisfaction on Psychological Well-Being through Emotional Labor

Job satisfaction can be classified into internal and external factors. Internal factors include a sense of accomplishment, completing work independently, work performance satisfied by the manager, work performance satisfied by the patient, work performance satisfied by the patient’s family, competence for the job, arriving at the patient’s home on time, and finishing the job on time with good quality. The effect of internal factors on psychological well-being through the mediating effect of deep acting is statistically significant. Although the effect of internal factors on psychological well-being through the mediating effect of surface acting demonstrated statistical significance in terms of the indirect effect, the coefficient of the indirect effect was less than for deep acting. 

In addition, the external factors of job satisfaction included benefits and systems regarding the company, the manager’s decision-making ability, the salary, workplace safety, workplace distance, flextime, job stability, and happiness reading the job. The effect of external factors on psychological well-being through the mediating effect of surface acting demonstrated statistically significance. However, the external factors did not show statistical significance in terms of the mediating effect of deep acting. It seems reasonable to posit that the effect of the external factors of job satisfaction on psychological well-being is driven by the mediating effect of surface acting.

Cross-culture differences in emotional labor have been reported [35]. For US service workers, a fairly strong correlation was discovered between display rules and surface acting leading to burnout, but this correlation was not observed in Chinese service workers [36]. This indicates that Chinese service workers are more likely to use deep acting to face their customers. These results may support our findings that deep acting plays a more important role in mediating job satisfaction and psychological well-being than surface acting for Taiwanese home-care workers. 

### 4.6. Limitations and Future Research

There are some limitations in this study. First, due to the self-reported questionnaire design used in this study, the common method of variance may have exaggerated the relationships that were found [37]. Future studies should aim to develop a more objective measurement regarding this topic. Second, we demonstrated that the internal factors of job satisfaction had an effect on psychological well-being through the mediating effect of deep acting. However, we did not investigate whether this could increase the employment rate of home-care workers. This research should be performed in the future. Thirdly, surface acting and deep acting are seen as effortful activities that demand the utilization of resources [38]. Employees may move to different emotional labor tactics that involve little emotional resource input. However, we did not consider the other emotional labor tactics in this study, which should be further investigated in the future. Fourthly, the study population is from Asia; therefore, the results may not be applicable to the general population. In future studies, other populations should be included for investigation to determine whether similar results can be obtained from them as well. Finally, although we have focused on the effect of job satisfaction on psychological well-being, other factors that may affect psychological well-being were not investigated in this study, such as leadership style and personality. 

### 4.7. Theoretical and Practical Implications

This study aimed to explore the psychology of home-care workers to develop and verify a theoretical frame work for understanding the relationships among emotional labor, job satisfaction, and psychological well-being. This study provides a more comprehensive understanding of the factors that influence the level of psychological well-being among home-care workers. The causal mediation analysis confirmed that emotional labor mediates the relationship between job satisfaction and psychological well-being among home-care workers in the south of Taiwan. In other words, emotional labor cannot be detached when investigating job satisfaction and psychological well-being phenomena in the psychology of home-care workers. We hope that our research will prompt an examination of the use of emotional labor as a possible practice in human resources to mediate the relationship between job satisfaction and psychological well-being.

## 5. Conclusions

In this study, we aimed to investigate the associations between job satisfaction, emotional labor, and psychological well-being for Taiwanese home-care workers. Our results show that job satisfaction is associated with emotional labor in which deep acting plays a more important role in emotional labor than surface acting. Moreover, our results demonstrate that emotional labor is associated with psychological well-being, in which both surface acting and deep acting are directly associated with psychological well-being, with a low level of association. In addition, our results demonstrate that job satisfaction was associated with psychological well-being in which the internal factors of job satisfaction should play a more important role in psychological well-being than the external factors of job satisfaction. Finally, we investigated the effect of job satisfaction on psychological well-being thorough emotional labor. Our results demonstrate the effect of job satisfaction on psychological well-being through the mediating effect of emotional labor for Taiwanese home-care workers. We found that the internal factors of job satisfaction are more important than the external factors affecting psychological well-being through the mediating effect of deep acting. These results could provide valuable information regarding Taiwanese home-care workers. The findings of this study hold a significant value in the context of managing emotional labor. We can increase the job satisfaction and psychological well-being of Taiwanese home-care workers by educating them to reassess situations. We hope that our research will prompt an examination of the use of emotional labor as a possible practice in human resources to mediate the relationship between job satisfaction and psychological well-being.

## Figures and Tables

**Table 1:** Characteristics of participants.

Variable	Mean ± SD (%)
*Country*	
Taiwan	305 (96.5%)
China	11 (3.5%)
Gender (females)	252 (79.7%)
Age	42.05 ± 12.15
*Marital status*	
married	201 (63.6%)
unmarried	78 (24.9%)
divorce	27 (8.5%)
widowed	10 (3.2%)
*Education*	
junior high school	20 (6.3%)
senior high school	99 (31.3%)
vocation high school	123 (38.9%)
university	70 (22.2%)
master	4 (1.3%)
Working year	5.64 ± 5.13
*Monthly salary (TWD)*	
<25,000	25 (7.9%)
25,001–35,000	160 (50.6%)
35,001–45,000	114 (36.1%)
45,001–55,000	14 (4.4%)
55,001–65,000	0 (0%)
>65,001	3 (1.0%)

TWD: New Taiwan dollars; SD: standard deviation.

**Table 2:** Pearson correlation analysis of job satisfaction, emotional labor, and psychological well-being.

	EJS	IJS	SA	DA	PWB
EJS	1	0.518 **	0.402 **	0.330 **	0.603 **
IJS		1	0.403 **	0.429 **	0.631 **
SA			1	0.512 **	0.507 **
DA				1	0.491 **
PWB					1

EJS: external factors of job satisfaction; IJS: internal factors of job satisfaction; SA: surface acting; DA: deep acting; PWB: psychological well-being. **: *p*-value < 0.001.

**Table 3:** Assessment of PLS-SEM model among emotional labor, job satisfaction, and psychological well-being.

Construct	Type	Items	Loadings	Cronbach’s Alpha	AVE	CR
Emotional labor	Deep acting	item1	0.717	0.856	0.636	0.897
		item2	0.788			
		item3	0.818			
		item4	0.839			
		item5	0.819			
	Surface acting	item1	0.746	0.77	0.594	0.853
		item2	0.836			
		item3	0.812			
		item4	0.68			
Job satisfaction	Internal factors	item1	0.768	0.919	0.64	0.934
		item2	0.837			
		item3	0.772			
		item4	0.884			
		item5	0.869			
		item6	0.795			
		item7	0.746			
		item8	0.712			
	External factors	item1	0.768	0.92	0.641	0.934
		item2	0.704			
		item3	0.833			
		item4	0.798			
		item5	0.833			
		item6	0.813			
		item7	0.807			
		item8	0.841			
Psychological well-being		item1	0.607	0.923	0.503	0.934
		item2	0.636			
		item3	0.656			
		item4	0.659			
		item5	0.629			
		item6	0.682			
		item7	0.706			
		item8	0.789			
		item9	0.738			
		item10	0.776			
		item11	0.755			
		item12	0.737			
		item13	0.747			
		item14	0.774			

AVE: average variance extracted; CR: composite reliability.

**Table 4:** Indirect effects of job satisfaction on psychological well-being.

Hypotheses	Coefficient	Standard Deviation	T Statistics	*p*-Value
IJS-SA-PWB (H9)	0.046	0.019	2.377	0.018 *
IJS-DA-PWB (H10)	0.053	0.020	2.683	0.007 *
EJS-DA-PWB (H11)	0.024	0.014	1.703	0.089
EJS-SA-PWB (H12)	0.046	0.016	2.797	0.005 *

EJS: external factors of job satisfaction; IJS: internal factors of job satisfaction; SA: surface acting; DA: deep acting; PWB: psychological well-being. *: *p*-value < 0.05.

## Data Availability

The dataset that supports the findings of this study is not openly available and it will be available from the corresponding author upon reasonable request.

## Appendix A

**Table A1:** The questionnaires were adopted in this study.

**Emotional Labor**	
Surface acting	
Item 1	When caring for individuals, not only do I appear happy on the outside but also feel happy inside.
Item 2	I try my best to overcome my negative emotions while working and provide friendly and kind service to the individuals under my care.
Item 3	Even if I know the individuals, I care for are being unreasonable, I am still able to empathize with their perspective and sincerely help them solve problems.
Item 4	Despite feeling tired, I strive to maintain appropriate emotions while working.
Deep acting	
Item 1	If I need to express a certain emotion (such as kindness or gentleness) in front of others, I would try my best to make it come from the heart rather than to fake it.
Item 2	When I am feeling down, I temporarily forget about it for the sake of work and maintain a positive attitude towards the individuals I care for.
Item 3	I work hard to cultivate empathy towards the individuals I care for.
Item 4	When faced with setbacks at work, I make an effort to remain calm and promptly work towards improving the situation of unease.
Item 5	I make an effort to understand the problems of the individuals I care for and provide solutions to ease their frustrations.
**Job Satisfaction** Internal factors	
Item 1	My job gives me a sense of accomplishment.
Item 2	I can complete tasks assigned by my supervisor independently.
Item 3	My supervisor recognizes my job performance.
Item 4	I receive recognition from service recipients for my job performance.
Item 5	I receive recognition from clients’ family members for my job performance.
Item 6	I am competent in the service items required for the job.
Item 7	I can arrive at the service recipient’s home on time.
Item 8	I can complete my work within the specified time and maintain good quality.
External factors	
Item 1	I am satisfied with the benefits and systems provided by the organization (such as labor insurance, health insurance, and bonuses).
Item 2	I am satisfied with my supervisor’s decision-making ability.
Item 3	I am satisfied with my current hourly wage and salary.
Item 4	I am satisfied with the safety of the work environment.
Item 5	I am satisfied with the location and distance of the assigned cases.
Item 6	I am satisfied with the flexibility of my work schedule.
Item 7	My current case load is stable.
Item 8	I am happy and satisfied with my current job.
**Psychological Well-being**	
Item 1	I like myself.
Item 2	I am deserving of love and trust.
Item 3	I do not feel alone.
Item 4	I love my life.
Item 5	The income from my job is sufficient to meet my needs, and I feel secure.
Item 6	I am capable of solving problems in my life.
Item 7	I am able to live the life I want.
Item 8	Overall, I am satisfied with my current job.
Item 9	I am respected at work.
Item 10	I receive recognition from clients and their families for my work.
Item 11	Seeing improvement in the individuals I care for makes my work meaningful and brings me joy.
Item 12	My work helps vulnerable individuals and benefits the society.
Item 13	My work brings happiness to others.
Item 14	My work performance consistently brings me a sense of accomplishment.
